# Over-the-Counter Painkillers and Evolutionary Mismatch

**DOI:** 10.3389/fpsyg.2019.00686

**Published:** 2019-04-02

**Authors:** Andrew C. Gallup

**Affiliations:** Psychology Program, SUNY Polytechnic Institute, Utica, NY, United States

**Keywords:** pain, acetamination, evolutionary mismatch, social pain, fever

People commonly reach for over-the-counter (OTC) pain medications to treat even the slightest physical discomfort, whether at work, at home, or on the go. Acetaminophen (paracetamol, Tylenol) tops the charts among popular painkillers, with estimates in the United States suggesting that nearly one in four adults consume a drug containing acetaminophen each week (Kaufman et al., 2002). As an analgesic, acetaminophen is quite effective in reducing mild to moderate physical pain and ailments, providing relief from headaches, and serving as a fever reducer. However, the accessibility and widespread use of this drug across the lifespan, even beginning in infancy (e.g., Infants' Tylenol), produces a mismatch with our evolved psychology to minimize pain and distress.

An evolutionary mismatch occurs when there is a major divergence between modern contexts and ancestral environments (reviewed by Li et al., 2018). Since changes to the environment drastically outpace the course of biological/genetic evolution, the conditions humans encounter in contemporary life are often vastly different from those of the hunter-gatherer lifestyles that shaped our brains and behavior. Thus, our psychological adaptations that evolved to solve recurrent problems of survival and/or reproduction during 99% of human evolutionary history are now activated by novel inputs of the modern world, which can lead to negative consequences for physical and psychological health (Li et al., 2018).

The experience of physical pain provides a clear example of a psychological adaptation. The unpleasantness of physical pain is highly adaptive and critical for survival. Pain draws our attention and serves to motivate responses to alleviate the discomfort. In ancestral conditions, this would have resulted in behavioral changes that functioned to reduce harm, minimize further damage, and prompt us to learn to avoid situations and actions that led to pain in the past. These benefits of physical pain are clearly witnessed among individuals unable to detect it, as those born with the rare condition of congenital insensitivity to pain often die in childhood due to the failure to notice injuries. Thus, there has been continuous selective pressure during the course of evolution to detect and reduce physical discomfort, and as a result most people possess a strong desire to avoid pain through behavioral changes that reduce exposure to harmful stimuli or events.

Within modern environments, however, individuals suffering from pain can now bypass behavioral changes and simply treat their symptoms with OTC medication. In fact, acetaminophen has been recommended as the first line of treatment for acute lower back pain (reviewed by Koes et al., 2010) and osteoarthritis (reviewed by Zhang et al., 2004), though more recent research has questioned the efficacy of acetaminophen treatment within chronic pain conditions (e.g., Machado et al., 2015; Ennis et al., 2016). People also frequently use painkillers to deal with injuries resulting from physical exercise or sporting competition. In one study of over 500 collegiate athletes, more than half (58%) reported regularly using either OTC or prescribed painkillers throughout the season to alleviate soreness from tough workouts and/or competition (Tricker, 2000). The continued dulling of physical pain from drugs, which undoubtedly feels good in the short-term, could produce maladaptive outcomes in the long-term if nothing is done to address the source of the pain itself. That is, routinely taking acetaminophen or similar OTC drugs to treat pain could exacerbate the physical damage, negatively impact recovery time, and even contribute to the acquisition of additional unnoticed injury or insult.

A distinct, yet somewhat similar, evolutionary mismatch comes from the fever reducing properties of acetaminophen and many other OTC pain medications. Research shows that acetaminophen and other OTC painkillers are effective and well-tolerated in treating febrile illness (McIntyre and Hull, 1996; Purssell, 2002), and as a result, many people reach for these drugs at the first signs of fever. Although unpleasant to endure, fever is our body's adaptive response to combat pathogens and the benefits appear to outweigh the costs (Harden et al., 2015). Taking antipyretics to reduce a fever halts the most effective natural defense against infection, and studies show that it can even produce detrimental health outcomes and increase mortality (Schulman et al., 2005; Eyers et al., 2010). Fever itself should thus not be considered a problem that needs to be remedied, unless of course, it is unusually high and/or contributing to other health problems, but rather it should viewed as an adaptive immune response that has been shaped by natural selection.

More recent and thought-provoking lines of research show that acetaminophen can produce further mismatch with our evolved psychology by even diminishing our sensitivity to social experiences that are interpreted as painful or distressing (reviewed by Ratner et al., 2018). As it turns out, the neurobiological systems that process physical and social pain overlap in the brain (Eisenberger et al., 2003), which gives credence to the saying “words hurt.” This neurological coupling makes sense from an evolutionary perspective given the critical importance of group membership and standing for survival and reproduction in our species. The biological response of social exclusion, for example, should be nearly as attention grabbing and aversive as a painful physical stimulus, and similarly motivate us to respond adaptively to mitigate damage.

In a radical set of studies, DeWall et al. (2010) found that after taking typical doses of acetaminophen daily for a period of 3 weeks, participants reported significantly lower levels of social pain in their daily lives and showed reduced cortical activity (i.e., dorsal anterior cingulate cortex and anterior insula) to social rejection scenarios produced in an experimental setting. Incredibly, subsequent studies have shown that even one-time use of acetaminophen in the laboratory can significantly reduce one's empathy for both physical and social pain experienced by others (Mischkowski et al., 2016). Furthermore, it has been found that acetaminophen blunts the conflict and pain of decision-making (DeWall et al., 2015) and dampens overall emotional perception and subjective arousal (Durso et al., 2015). Collectively, these effects could produce a variety of unintended and maladaptive psychological outcomes in the real world. For example, individuals that routinely take acetaminophen to treat physical pain or headaches in their daily lives may fail to adequately process negative interpersonal interactions, lack typical and appropriate responses to affective cues, show reduced empathy and affiliation toward partners and close friends in times of distress, and suffer from impaired forms of judgement or decision-making.

Future research should aim to examine the full range of psychoactive effects produced by acetaminophen and other OTC painkillers. Based on evidence supporting a general cortical dampening produced by acetaminophen (DeWall et al., 2010), one potentially fruitful area to examine would be whether this drug also modulates the processing of subcortical and more prewired psychological adaptations. Among primates, for example, a group of neurons in the pulvinar nucleus of the thalamus has been shown to selectively fire in response to images of snakes (Van Le et al., 2013), and this occurs even among individuals reared in captivity that have had no prior experience with these dangerous animals. Determining how acetaminophen alters the psychological processing of distressing stimuli and situations that vary in ancestral and evolutionary relevance, and evoke distinct patterns of neural activation within the brain, would be important for determining the magnitude and scope of the blunting effects produced by this drug. This type of research could also provide a more systematic approach to assessing how OTC painkillers contribute to mismatches with our evolved psychology.

To date, research examining the psychological effects of acetaminophen and other OTC painkillers is still in its infancy (Ratner et al., 2018), so it remains unknown how the typical consumption of these drugs influence the real world interactions and varied interpersonal relationships among the millions of the people taking them on a daily basis. While the analgesic properties of these drugs appear quite effective and well-tolerated, the potential negative consequences abound, and thus we should be wary of frequent or routine usage of these medications. A little pain and distress, whether physical or social, is good for us, and we should therefore embrace its abrasiveness.

## Author Contributions

The author confirms being the sole contributor of this work and has approved it for publication.

### Conflict of Interest Statement

The author declares that the research was conducted in the absence of any commercial or financial relationships that could be construed as a potential conflict of interest.
